# Positive Childhood Experiences, Suicide Cognitions, Subjective Happiness, and Mental Well-Being in Young Adults: A Half-Longitudinal Serial Mediation Study

**DOI:** 10.1007/s11126-025-10162-6

**Published:** 2025-05-28

**Authors:** Ömer Faruk Akbulut

**Affiliations:** Quality Coordinatorship Unit, Afyonkarahisar Health Sciences University, Afyonkarahisar, 03030 Türkiye

**Keywords:** Positive childhood experiences, Suicide cognitions, Subjective happiness, Mental well-being, Longitudinal serial mediation study

## Abstract

Childhood experiences play an important role in shaping individuals’ well-being. The relationships between childhood experiences, suicide cognitions, happiness, and mental well-being have been examined with cross-sectional methods. However, the lack of any longitudinal study in which these variables were examined together made it necessary to conduct this study. In this study, we tested whether suicide cognitions and subjective happiness serially mediate the relationship between positive childhood experiences and mental well-being. After matching the data obtained from the first (T1) and second waves (T2) of the study, the final sample consisted of 234 Turkish young adults (M = 20.70 years, SD = 2.22). Participants completed the Positive Childhood Experiences Scale, The Brief Suicide Cognitions Scale, Subjective Happiness Scale, and Warwick-Edinburgh Mental Well-Being Scale Short Form. For this purpose, a study was conducted in two separate time waves and structural equation modeling was used to test longitudinal serial mediation between variables. According to the results, suicide cognitions and subjective happiness play a serial mediating role in the longitudinal relationship between positive childhood experiences and mental well-being. These findings provide both theoretical insight into the cognitive and emotional mechanisms linking early childhood experiences to mental well-being and practical implications for mental health promotion in young adulthood. The findings of the study were discussed and interpreted in detail in the light of the relevant literature.

**Supplementary Information:**

The online version contains supplementary material available at 10.1007/s11126-025-10162-6.

## Introduction

Childhood experiences play an important role in shaping individuals’ well-being and mental health across the lifespan [7]. To date, a significant body of research has emphasized the harmful effects of adverse childhood experiences such as trauma, neglect, and family conflict [22, 27, 30, 48]. However, there is growing interest in understanding how positive childhood experiences contribute to individuals’ well-being [17, 40]. Positive childhood experiences, characterized by supportive relationships, social connectedness, emotional safety, and opportunities for development, are associated with multiple indicators of mental well-being in adulthood [45]. Research shows that individuals with positive childhood experiences exhibit higher resilience, happiness, and well-being [14, 16, 44]. In addition, positive childhood experiences enable individuals to develop emotion regulation skills and use healthy coping strategies [42]. Despite these well-documented associations, the mechanisms underlying the relationship between positive childhood experiences and mental well-being are poorly understood. Given the complexity of the effects of childhood experiences, it is likely that various cognitive and emotional processes mediate this relationship [63]. Examining these mediating effects may contribute to a deeper understanding of the long-term psychological effects of positive childhood experiences. In this context, this study examines the relationship between positive childhood experiences and mental well-being in the context of suicide cognitions and subjective happiness.

### Positive Childhood Experiences and Mental Well-Being

Positive childhood experiences are early supportive and developmental experiences that shape an individual’s well-being and contribute to the development process [6, 55]. These experiences are associated with long-term developmental outcomes by increasing individuals’ psychological resilience [38]. The role of positive childhood experiences on mental well-being is addressed within the framework of various theoretical approaches [17]. Attachment Theory [8], one of these theoretical approaches, emphasizes that secure early relationships with caregivers form the basis of healthy emotional and social development. Bonds based on warmth, sensitivity, and emotional harmony established with the caregiver in the early period have a protective effect on mental well-being later in life [64]. Another theoretical approach, Ecological Systems Theory [10], emphasizes the role of various environmental factors, especially family and peers, in influencing developmental processes. The Developmental Psychopathology (DP) Theory [62] talks about how biological, psychological, and environmental factors interact. It also says that early positive experiences help protect emotional health. The Resilience Theory places a similar emphasis. According to this theory [59], positive childhood experiences act as a protective factor for individuals’ mental well-being, promote adaptive responses to challenges, and increase psychological resilience. Collectively, these theoretical frameworks underscore that early positive experiences contribute to mental well-being by fostering emotional security, resilience, and adaptive functioning across the lifespan.

Positive childhood experiences can contribute to the development of mental well-being by promoting emotional safety, self-regulation, and adaptive coping strategies [23]. Indeed, these positive experiences support the five core components (positive emotions, engagement, relationships, meaning, and achievement) that contribute to the development of mental well-being in line with the PERMA model [2]. Positive childhood experiences promote emotional security, autonomy, social bonds, purpose, and resilience [55]. By supporting these five core components, positive childhood experiences serve as a protective factor against mental health problems and contribute to long-term well-being [3]. Existing research shows that individuals with positive childhood experiences have lower levels of depression, anxiety, and stress, and higher life satisfaction and social functioning [23, 34, 44]. While these effects are well documented, it remains important to explore the mechanisms underlying the relationship between positive childhood experiences and mental well-being. These theoretical perspectives not only highlight the importance of positive childhood experiences for well-being, but also help explain how such experiences may influence mental well-being through key mechanisms such as suicide cognitions and subjective happiness. In this context, this study examines suicide cognitions and subjective happiness as potential mediating variables.

### Serial Mediation of Suicide Cognitions and Subjective Happiness

Positive childhood experiences serve as a protective factor against psychological distress [58]. Different theoretical approaches [59, 62] suggest that early positive experiences contribute to the development of an individual’s cognitive and emotional resources and support adaptive coping strategies and psychological resilience. In this respect, positive childhood experiences can serve as a buffer against stress, depression, and suicide risk later in life [3]. The interpersonal theory of suicide highlights that feelings of perceived burdensomeness and thwarted belongingness are key risk factors for suicidal ideation [19]. In this context, positive childhood experiences may reduce the likelihood of individuals developing maladaptive thought patterns related to suicide by increasing feelings of connectedness, self-worth, and emotional security [33]. Current research suggests that individuals with a history of supportive relationships, nurturing family environments, and positive social interactions during childhood exhibit lower levels of suicide cognitions and behaviors in adulthood [9, 14, 32]. Suicide cognitions, which encompass thoughts related to hopelessness, perceived burdensomeness, and the desire for escape from psychological pain, are closely linked to overall mental well-being [28]. The cognitive model of suicide states that negative self-schemas and maladaptive cognitive patterns may increase the risk of suicide and may be a risk factor for mental well-being [52]. Previous research shows that suicide cognitions are associated with lower well-being, resilience, and social functioning [4, 11]. Therefore, suicide cognitions may be a mediating factor in the relationship between positive childhood experiences and mental well-being.

Another psychological mechanism that may bridge positive childhood experiences and mental well-being is subjective happiness. Secure and supportive relationships in early childhood contribute to higher life satisfaction and happiness in adulthood [7]. The broaden-and-build theory of positive emotions suggests that positive childhood experiences can expand an individual’s cognitive and emotional resources and increase resilience and the ability to find meaning in life [31]. Indeed, limited empirical research suggests that individuals who report positive childhood experiences have higher levels of subjective happiness [29, 35]. Increased subjective happiness also positively affects individuals’ mental well-being [54]. Subjective happiness is a concept that expresses the extent to which an individual finds his/her life happy and satisfying [43]. In this respect, subjective happiness is an important component of mental well-being, which reflects an individual’s overall life satisfaction and sense of positivity [36]. Existing research emphasizes the strong relationships between subjective happiness and mental well-being [25, 39]. In addition, research shows that individuals with higher levels of subjective happiness experience lower levels of stress, anxiety, and depression [50, 57]. Such factors may contribute to individuals’ mental well-being. Therefore, we can say that subjective happiness may mediate the relationship between positive childhood experiences and mental well-being.

In addition, suicide cognitions can affect individuals’ subjective happiness [51]. Suicide cognitions, which are associated with maladaptive thoughts about oneself and one’s life, are a risk factor for mental well-being [5]. Suicide cognitions can limit cognitive flexibility and increase feelings of hopelessness [13]. Such thoughts may cause the individual’s adaptive coping strategies to be inadequate in the face of challenging life events [53]. In this context, suicide cognitions can reduce individuals’ subjective happiness. Previous research has shown that higher levels of suicidal ideation are significantly associated with lower levels of subjective happiness [12, 46]. Therefore, suicide cognitions and subjective happiness are posited to have a serial mediating role in the relationship between positive childhood experiences and mental well-being.

### The Present Study

The existing literature emphasizes that positive childhood experiences have an important role in shaping individuals’ mental well-being. While previous research has shown that positive childhood experiences serve as a protective factor against a variety of mental problems, there is limited empirical evidence on the mechanisms through which these effects emerge over time [41, 58]. In particular, the serial mediation pathways linking positive childhood experiences to mental well-being through suicide cognitions and subjective happiness have been understudied. To address this gap, this study examines the direct and indirect effects of positive childhood experiences on mental well-being among young adults using a quasi-longitudinal serial mediation model. Childhood experiences are a concept that has long-term effects on individuals’ lives [56]. Therefore, it is critical to longitudinally examine this concept. The findings of this study are expected to provide valuable insights into the role of positive childhood experiences in individuals’ mental well-being. In this context, the purpose of this study is to examine the serial mediation roles of suicide cognitions and subjective happiness in the relationship between positive childhood experiences and mental well-being through longitudinal mediation analysis in a sample of young adults. Consequently, the study addresses the following research questions:

RQ1. Does suicide cognitions mediate the longitudinal relationship between positive childhood experiences and mental well-being?

RQ2. Does subjective happiness mediate the longitudinal relationship between positive childhood experiences and mental well-being?

RQ3. Do suicide cognitions and subjective happiness jointly function as serial mediators in the longitudinal relationship between positive childhood experiences and mental well-being?

## Method

### Procedure and Participants

This longitudinal study was conducted at four-month intervals. The participant form was completed online. This study complied with the ethical standards set forth in the 1964 Helsinki Declaration and its subsequent updates. Professional ethical principles were adhered to throughout the research. Then, the research data was collected using a web-based form. The link to the online form was sent to the participants via the authors’ social media accounts (Whatsapp, Twitter, Instagram, etc.). Prior to participating in the study, each participant provided informed consent. Participants participated in the study voluntarily, and no fee was paid to the participants throughout the research. The online form was designed so that participants could withdraw at any time and resubmit once all questions had been answered.

The longitudinal data included 292 participants in time one and 264 participants in time two. The questionnaires for the matching study included the last three letters of the mother’s name, the first three letters of the father’s name, and a section with the pseudonym. The final sample included 234 adult participants who participated in both waves. Possible reasons for data loss across the two waves included participant dropout due to changes in contact information or personal circumstances, as well as incomplete or invalid questionnaires that could not be matched. The final sample included 190 females (%81.2) and 44 males (%18.8). Participants’ ages ranged from 18 to 31 years (Mage = 20.70, SD = 2.22).

### Measures

#### Positive Childhood Experiences Scale

The scale was developed by Bethell et al. [6] to assess the positive experiences of individuals before the age of eighteen. The Turkish adaptation of the scale was carried out by [21]. The scale is a seven-item scale (e.g., ‘How much of your childhood did you have an adult in your home who made you feel safe and protected?’) that assesses positive childhood experiences. Items are rated on a 5-point scale from 1 (never) to 5 (always). Higher scores on the scale indicate greater levels of positive childhood experiences. The scale is unidimensional. The Turkish version of the scale exhibits robust psychometric properties, including very good construct validity (RMSEA = 0.07, SRMR = 0.07, GFI = 0.97, CFI = 0.97, NFI = 0.96, and IFI = 0.96), and excellent reliability (α = 0.78). In this study, the reliability of the scale was acceptable at both time points: for T1 (ω = 0.65, α = 0.66, λ6 = 0.67) and for T2 (ω = 0.68, α = 0.69, λ6 = 0.70).

#### The Brief Suicide Cognitions Scale

The scale was developed by Rudd ve Bryan (2021) to assess the suicidal thoughts and beliefs. The Turkish adaptation of the scale was carried out by [4]. The scale is a six-item scale (e.g., ‘I can’t deal with my problems anymore.’) that assesses suicide cognitions. Items are rated on a 5-point scale from 1 (never) to 5 (always). Higher scores on the scale indicate greater level of suicide cognitions. The scale is unidimensional. The Turkish version of the scale exhibits robust psychometric properties, including very good construct validity (RMSEA = 0.08, SRMR = 0.03, GFI = 0.98, CFI = 0.98, and TLI = 0.96), and excellent reliability (α = 0.88). In this study, the reliability of the scale was acceptable at both time points: for T1 (ω = 0.88, α = 0.88, λ6 = 0.88) and for T2 (ω = 0.88, α = 0.88, λ6 = 0.88).

#### Subjective Happiness Scale

This scale, developed by Lyubomirsky ve Lepper (1999), was adapted to Turkish culture by [1]. The last item of the four-item scale (e.g., “Some people are usually very happy, enjoying everything, no matter what is going on.” To what extent does such a statement describe you?”) is scored reversely. After the reverse item is arranged, a total score is obtained from the scale, which is scored in a seven-point rating form. Items are rated on a seven-point scale from 1 (It’s not suitable) to 7 (completely appropriate). As the scores that can be obtained from the scale increase, the subjective happiness level of individuals increases. The scale is unidimensional. The Turkish version of the scale exhibits robust psychometric properties, including very good construct validity (RMSEA = 0.00, SRMR = 0.01, AGFI = 0.99, GFI = 1.00, NFI = 0.99, CFI = 1.00 and RFI = 0.98), and excellent reliability (α = 0.86). In this study, the reliability of the scale was acceptable at both time points: for T1 (ω = 0.84, α = 0.84, λ6 = 0.82) and for T2 (ω = 0.82, α = 0.81, λ6 = 0.80).

#### Warwick-Edinburgh Mental Well-Being Scale Short Form

The scale was developed by Tennant et al. [60] to determine the mental well-being levels of individuals. The Turkish adaptation of the scale was carried out by Demirtaş and Baytemir [26]. The scale is a seven-item scale (e.g., ‘Dealing with problems well getting out.’) that assesses mental well-being. Items are rated on a 5-point scale from 1 (never) to 5 (always). Higher scores on the scale indicate greater levels of mental well-being. The scale is unidimensional. The Turkish version of the scale demonstrates robust psychometric properties, including very good construct validity (RMSEA = 0.06, SRMR = 0.03, CFI = 0.99, NFI = 0.97, GFI = 0.97, and AGFI = 0.94) and excellent reliability (α = 0.84). In this study, the reliability of the scale was acceptable at both time points: for T1 (ω = 0.84, α = 0.84, λ6 = 0.84) and for T2 (ω = 0.84, α = 0.83, λ6 = 0.82).

### Data Analysis

In this study, firstly the normality test of the data was examined. After ensuring normal distribution, descriptive statistics, correlation coefficients, and different reliability values (McDonald omega, Cronbach alpha, and Guttman lambda) were calculated. Using structural equation modeling, we investigated whether suicide cognitions and subjective happiness mediate the longitudinal serial relationship between positive childhood experiences and mental well-being. Positive childhood experiences are the model’s independent variable. Suicide cognitions and subjective happiness are serial mediators. Mental well-being is the dependent variable. In the analyses of this study, parceling technique was used. This technique should be preferred to reduce measurement errors when using one-factor scales [49]. Therefore, in the study, parceling method was used for positive childhood experiences, suicide cognitions, subjective happiness, and mental well-being variables in this study. Apart from this, the model’s goodness of fit can be measured using various indices, such as χ2 to degrees of freedom, Comparative Fit Index (CFI), Tucker-Lewis Index (TLI), Normed Fit Index (NFI), Incremental Fit Index (IFI), Goodness of Fit Index (GFI), and Standardized Root Mean Square Residual (SRMR). According to Kline [37], CFI, IFI, TLI, NFI, and GFI values of 0.90 or higher are generally considered adequate model fit, whereas values of 0.95 or higher are preferred for good model fit. Furthermore, Kline [37] suggests that an SRMR cut-off value of 0.08 or lower indicates a satisfactory model fit. The longitudinal mediation model was tested using 5000 bootstrap samples. The significance of the direct and indirect effects was assessed based on 95% bias-corrected bootstrap confidence intervals. An effect was considered statistically significant if the confidence interval did not include zero. Lastly, gender and age were included in the model as control variables. These calculations were performed using SPSS 25.0, JASP 0.18.3.0, AMOS Graphics 24.0 statistical package programs.

## Results

### Preliminary Analyses

All findings obtained within the scope of preliminary analyses are shown in Table 1. First, normality values were examined, then descriptive statistics and reliability values of the variables were calculated. Additionally, correlation analysis was performed for the relationships between variables. The analysis revealed that the variables exhibited a normal distribution and exhibited sufficient reliability. Additionally, all variables were found to be statistically significant with each other. Positive childhood experiences at T1-2 have a significant negative correlation with suicide cognitions at T1-2. At T1-2, however, it has a significant positive correlation with subjective happiness and mental well-being. Suicide cognitions at T1-2 has a significant negative relationship with subjective happiness and mental well-being. Furthermore, at T1-2, there are strong positive relationships between subjective happiness and mental well-being.

**Table 1:** Descriptive Statistics, Reliabilities, normality Assumptions, and correlations for the study variables

Variable	1	2	3	4	5	6	7	8
1. PCEs **T1**	–							
2. PCEs **T2**	0.698^**^	–						
3. Suicide cognitions **T1**	− 0.491^**^	− 0.341^**^	–					
4. Suicide cognitions **T2**	− 0.415^**^	− 0.463^**^	0.616^**^	–				
5. Subjective happiness **T1**	0.431^**^	0.272^**^	− 0.532^**^	− 0.477^**^	–			
6. Subjective happiness **T2**	0.376^**^	0.341^**^	− 0.390^**^	− 0.557^**^	0.653^**^	–		
7. Mental well-being **T1**	0.515^**^	0.379^**^	− 0.621^**^	− 0.510^**^	0.579^**^	0.452^**^	–	
8. Mental well-being **T2**	0.490^**^	0.534^**^	− 0.396^**^	− 0.626^**^	0.583^**^	0.684^**^	0.569^**^	–
Mean	24.74	25.03	11.43	11.00	18.24	18.38	24.52	24.91
SD	4.43	4.25	4.27	3.93	4.92	4.58	4.95	4.58
Skewness	-0.19	-0.26	0.99	1.04	-0.56	-0.65	-0.30	-0.25
Kurtosis	0.36	0.34	1.68	2.32	0.01	0.66	0.23	0.44
McDonald’s ω	0.65	0.68	0.88	0.88	0.84	0.82	0.84	0.84
Cronbach α	0.66	0.69	0.88	0.88	0.84	0.81	0.84	0.83
Guttman’s λ6	0.67	0.70	0.88	0.88	0.82	0.80	0.84	0.82

Note. * *p* <.05, ** *p* <.01; PCEs: Positive childhood experiences

### Structural Equation Modeling

Following the completion of the preliminary analyses, a two-stage structural equation modeling analysis was performed using the AMOS Graphics statistical package program. Then, the significance of the mediating roles was tested using the bootstrapping method. In the first stage, different measurement models were evaluated. The measurement model consists of four latent variables—positive childhood experiences, suicide cognitions, subjective happiness, and mental well-being—represented by a total of eight observed variables. The first wave measurement model was good-fitted to the data (χ2 = 44.275, df = 14, χ2/df = 3.16, CFI = 0.974, TLI = 0.948, NFI = 0.963, IFI = 0.974, GFI = 0.954, SRMR = 0.030) and the second wave (χ2 = 20.849, df = 14, χ2/df = 1.48, CFI = 0.994, TLI = 0.989, NFI = 0.981, IFI = 0.994, GFI = 0.978, SRMR = 0.021). Moreover, all factor loadings are between 0.702 and 0.913 and are significant. This means that the observed variables adequately manipulate the latent variables.

**Fig. 1:**
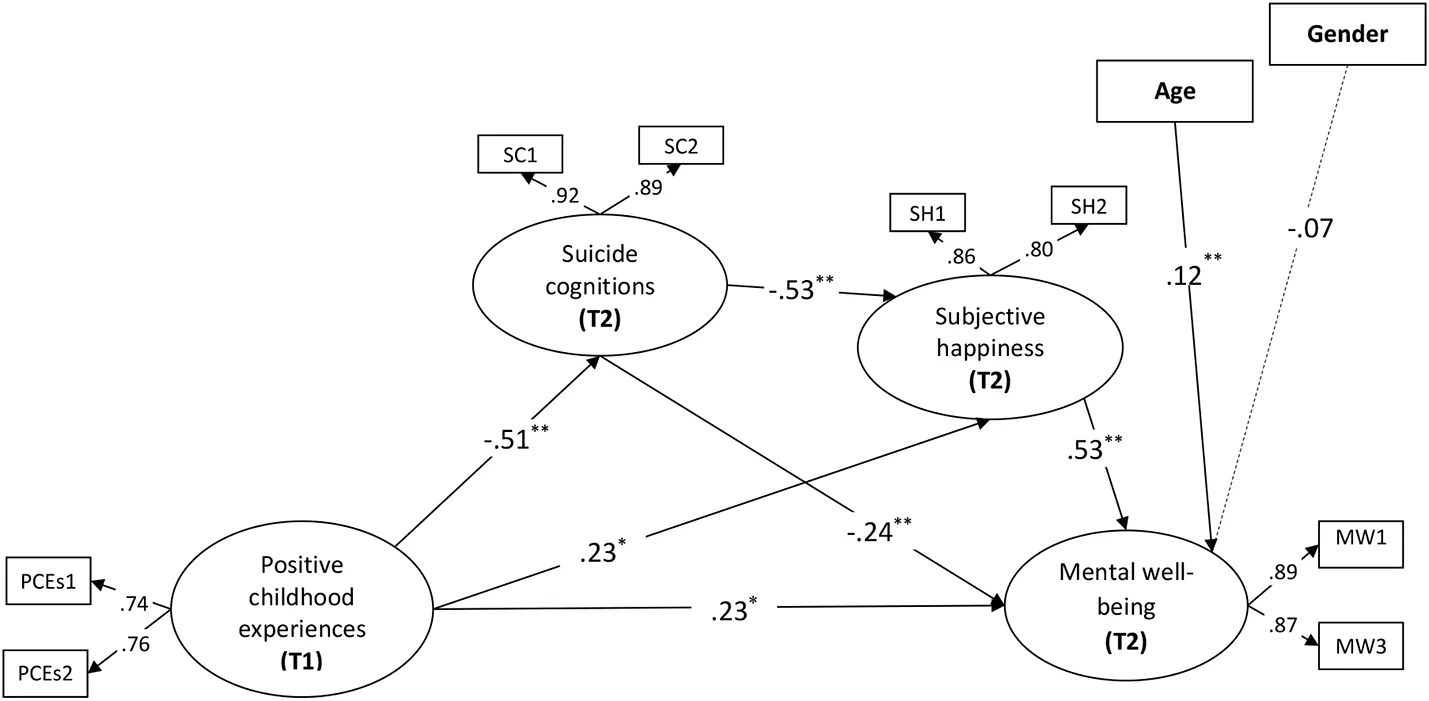
Structural equation modeling for the longitudinal serial mediation model. *p <.01, **p <.001, PCEs: Parcel of positive childhood experiences, SC: Parcel of suicide cognitions, SH: Parcel of subjective happiness, MW: Parcel of mental well-being; T1: Time 1; T2: Time 2

Following the measurement model, the longitudinal structural model was tested. The model was designed to examine whether suicide cognitions and subjective happiness play a serial mediating role in the longitudinal relationship between positive childhood experiences and mental well-being. As a result of the analysis, it was determined that all paths in the longitudinal structural model were statistically significant and the fit index values were at a sufficient level: χ² (28, *N* = 234) = 45.745, *p* <.05; χ²/df = 1.634; CFI = 0.983; TLI = 0.973; NFI = 0.959; GFI = 0.965; IFI = 0.984; RMSEA = 0.052; SRMR = 0.051 (see Fig. 1). In the structural model, positive childhood experiences significantly predicted suicide cognitions (β = − 0.51, *p* <.001), subjective happiness (β = 0.23, *p* <.01), and mental well-being (β = 0.23, *p* <.01). Suicide cognitions negatively predicted subjective happiness (β = − 0.53, *p* <.001), which in turn positively predicted mental well-being (β = 0.53, *p* <.001). All path coefficients were statistically significant. This finding suggests that positive childhood experiences have a significant effect on suicide cognitions, subjective happiness, and mental well-being. Moreover, suicide cognitions and subjective happiness have significant effects on mental well-being.

The significance of the mediating roles obtained was tested using the bootstrapping method. In this context, Table 2 presents the standardized coefficients for the direct and indirect effects of the variables. As As shown in Table 2, the direct effect of positive childhood experiences on mental well-being was significant (β = 0.233, 95% CI [0.074, 0.400]). In terms of indirect effects, the pathway through suicide cognitions (β = 0.144, 95% CI [0.042, 0.277]), the pathway through subjective happiness (β = 0.140, 95% CI [0.011, 0.330]), and the serial mediation path through both mediators (β = 0.162, 95% CI [0.089, 0.293]) were all statistically significant. The total indirect effect was also significant (β = 0.388, 95% CI [0.261, 0.525]), indicating a robust serial mediation effect. Since the 95% bootstrap confidence intervals did not include zero, the mediation effects can be considered statistically significant. According to conventional guidelines [20], values around 0.10 are considered small, around 0.30 moderate, and 0.50 or above large. In the current model, the direct effect of positive childhood experiences on mental well-being represented a moderate effect (β = 0.233), while the indirect effects through suicide cognitions (β = 0.144) and subjective happiness (β = 0.140) fell within the small-to-moderate range. The serial mediation path (β = 0.162) also indicated a small-to-moderate impact.

**Table 2:** Effect of serial mediation model

		95%CI	
Path	Coefficient	*LL*	*UL*
**Direct Effect**			
PCEs ◊ Mental well-being	0.233	0.074	0.400
**Indirect Effects**			
PCEs ◊ Suicide cognitions ◊ Mental well-being	0.144	0.042	0.277
PCEs ◊ Subjective happiness ◊ Mental well-being	0.140	0.011	0.330
PCEs ◊ Suicide cognitions ◊ Subjective happiness ◊ Mental well-being	0.162	0.089	0.293
**Total indirect effect**	0.388	0.261	0.525

Note. PCEs positive childhood experiences; CI confidence interval; LL lower limit; UL upper limit

## Discussion

This study examined the longitudinal serial mediating role of suicide cognitions and subjective happiness in the association between positive childhood experiences and mental well-being. Within this framework, the key findings were as follows: (1) suicide cognitions longitudinal mediated the relationship between positive childhood experiences and mental well-being, and (2) subjective happiness longitudinal mediated the relationship between positive childhood experiences and mental well-being, and (3) the association between positive childhood experiences and mental well-being was longitudinal serially mediated by both suicide cognitions and subjective happiness. Consequently, the study provided answers to the research questions. The results indicated the serial mediating roles of suicide cognitions and subjective happiness. The findings are discussed in more detail below.

The first finding of the study addresses the first research question (RQ1). Specifically, higher levels of positive childhood experiences were associated with an decreased suicide cognitions, which, in turn, predicted higher levels of mental well-being. This finding is consistent with previous research showing that positive childhood experiences function as a protective factor against psychological distress [17, 44]. Theoretical explanations suggest that early positive experiences contribute to the development of cognitive and affective resources [59, 62]. These benefits can improve individuals’ adaptive coping strategies [23]. Therefore, positive childhood experiences may have a protective role against stress, depression, and suicide risk [34]. Previous research suggests that individuals with positive childhood experiences exhibit lower suicide cognitions and behaviors [3, 9]. The findings of this study suggest that suicide cognitions may also affect individuals’ mental well-being. Moreover, the cognitive model of suicide suggests that maladaptive cognitive patterns and self-schemas may increase suicide risk and negatively affect mental well-being [52]. Indeed, current research shows that suicide cognitions predict lower levels of resilience, happiness, and well-being [4, 15]. Overall, these findings highlight the mediating role of suicide cognitions in the link between early positive experiences and mental well-being.

The findings of this study suggest that subjective happiness longitudinally mediates the relationship between positive childhood experiences and mental well-being. Safe and supportive interactions early in life contribute to greater happiness in adulthood [7]. The broaden-and-build theory of positive emotions suggests that positive childhood experiences increase psychological resilience by expanding individuals’ cognitive and emotional resources [31]. Consistent with this framework, prior studies have demonstrated that individuals who report higher levels of positive childhood experiences tend to exhibit greater subjective happiness [29, 44]. Increased subjective happiness can also positively affect the well-being of individuals [54]. As a matter of fact, subjective happiness is considered an important indicator of mental well-being [36]. This context suggests that subjective happiness could significantly impact mental well-being. Existing research emphasizes the strong relationship between subjective happiness and mental well-being [25, 61]. These studies specifically emphasize that individuals with higher levels of happiness tend to experience lower levels of stress, anxiety, and depression. This contributes to their mental well-being. These findings address (RQ2), indicating that subjective happiness serves as a longitudinal mediator in the relationship between positive childhood experiences and mental well-being.

Finally, the third finding of the study addresses the first research question (RQ3). This study found that the longitudinal serial mediating role of “suicide cognitions– subjective happiness” may explain the association between positive childhood experiences and mental well-being. Suicidal cognitions are a risk factor for mental well-being as they are associated with maladaptive thoughts about oneself and one’s life [19]. In this context, suicide cognitions can shape individuals’ subjective happiness [51]. These maladaptive cognitions may limit cognitive flexibility and increase feelings of hopelessness [13]. Suicide cognitions are considered a risk factor that reduces subjective happiness [24]. Research shows that suicide cognitions are linked to lower levels of subjective happiness [18, 47]. Therefore, these findings support the conceptualization of positive childhood experiences as an indirect serial mediator of mental well-being.

### Limitations

Some limitations should also be taken into consideration when evaluating the results of this research. First, the data were collected using self-report scales. Although voluntary, the use of self-report scales may cause social desirability errors. Secondly, collecting data online in longitudinal studies may lead to matching problems with pseudonym information, which can be considered another limitation. Future studies may prefer different data collection methods. Thirdly, the research data were collected and analyzed at four-month intervals. The half-longitudinal design of the study and the absence of a third wave of data limited our capacity to determine the exact causality of the relationships between variables. In future studies, researchers can retest the model with more waves of data and longer intervals between waves. Fourth, this study addresses the long-term relationships between positive childhood experiences, suicide cognitions, subjective happiness, and mental well-being. Future research could develop more comprehensive models to support the effects of positive childhood experiences by considering other possible variables that may be involved in this relationship. Finally, in this study, data were collected only from Turkish participants through convenience sampling. Therefore, future studies involving different cultural groups are recommended to increase the generalizability of the findings. Moreover, since the participants were recruited via social media platforms, the possibility of sampling bias should be acknowledged, as the sample may not fully represent the broader young adult population. We suggest future research delve deeper into unraveling these links.

### Implications

Positive child experiences play a critical role in individuals’ well-being. Positive experiences early in life contribute to reduced suicide cognitions, predicting higher levels of subjective happiness and ultimately enhancing mental well-being. Given that positive childhood experiences support the development of cognitive and emotional resources, it is crucial to promote open and supportive communication between children and their caregivers from an early age. In this context, the findings highlight the importance of developing psycho-educational programs for parents. Such programs should focus on promoting secure attachment, developing emotional regulation skills, and supporting effective parent-child communication. Furthermore, the longitudinal design of this study underlines the long-term impact of positive childhood experiences on individuals and emphasizes the necessity of early interventions. Future research, longitudinal studies with the participation of different cultural groups, may help us understand the effects of positive childhood experiences on individuals from a broader perspective. Lastly, although this study focused on young adults, the findings underline the lasting impact of positive childhood experiences and suggest that school-based interventions during childhood and adolescence may help strengthen resilience and support future mental well-being.

## Conclusion

The results of this research suggest that suicide cognitions and subjective happiness mediate the relationship between positive childhood experiences and mental well-being in a longitudinal series. This study empirically supports previous theoretical explanations by establishing a link between positive childhood experiences and mental well-being. The longitudinal design of the study provided a clearer picture of the dynamics of the relationship between the variables over time. These findings emphasize the critical role of positive childhood experiences in individuals’ mental well-being. Early supportive environments appear to enhance well-being and reduce the risk of maladaptive cognitive patterns such as suicide cognitions. Given the implications of these findings, promoting positive childhood experiences through family, educational, and community-based interventions may contribute to better mental well-being in adulthood. Finally, cross-cultural research on the subject may make a unique contribution to the literature.

## Electronic Supplementary Material

Below is the link to the electronic supplementary material.

Supplementary Material 1

## Data Availability

Data will be available on request.

## Declarations

### Pre-registration Statement

This study was not pre-registered.
