# Supplementary material for: Positive Childhood Experiences, Suicide Cognitions, Subjective Happiness, and Mental Well-Being in Young Adults: A Half-Longitudinal Serial Mediation Study
Source: Psychiatr Q. 2025 May 28;97(3):663–78. doi: 10.1007/s11126-025-10162-6 (PMC13518451; doi:10.1007/s11126-025-10162-6)
Supplement: Supplementary file 1 [file 11126_2025_10162_MOESM1_ESM.docx]

**Disclosure of Interest Form**

**Conflict of Interest.** No conflict of interest exists for this manuscript for any of the author.
